# Catastrophic Antiphospholipid Syndrome: A Review of Current Evidence and Future Management Practices

**DOI:** 10.7759/cureus.69730

**Published:** 2024-09-19

**Authors:** Ayoyimika O Okunlola, Temitope O Ajao, Mwila Sabi, Olayinka D Kolawole, Osasere A Eweka, Abbas Karim, Toluwani E Adebayo

**Affiliations:** 1 Internal Medicine, United Lincolnshire Hospitals NHS trust, Lincolnshire, GBR; 2 General and Acute Medicine, United Lincolnshire Hospitals NHS Trust, Lincolnshire, GBR; 3 Anaesthetics and ICU, United Lincolnshire Hospitals NHS Trust, Lincolnshire, GBR; 4 Internal Medicine, Hull University Teaching Hospitals NHS Trust, Hull, GBR; 5 Family Medicine, United Lincolnshire Hospitals NHS Trust, Lincolnshire, GBR; 6 Emergency Medicine, United Lincolnshire Hospitals NHS Trust, Lincolnshire, GBR; 7 General Medicine, Bowen College of Health Sciences, Iwo, NGA

**Keywords:** anti coagulation, antiphospholipid antibody syndrome, blood clots, catastrophic antiphospholipid syndrome, multiorgan system failure, thrombosis

## Abstract

Antiphospholipid syndrome (APS) is an autoimmune disorder characterized by blood clots and pregnancy complications due to antiphospholipid antibodies. Catastrophic APS (CAPS), a severe variant, leads to multiorgan failure and is often fatal. Pathogenesis involves antiphospholipid antibodies, particularly anti-beta-2-glycoprotein I (aβ2GPI), which trigger endothelial cell (EC) activation, cytokine release, and a prothrombotic state. Infections, surgeries, and other triggers can precipitate CAPS, leading to widespread microthromboses and systemic inflammatory responses. CAPS predominantly affects younger patients and those with systemic lupus erythematosus (SLE), with a high mortality rate, though recent treatment advances have improved survival. Diagnosing CAPS involves identifying clinical manifestations, including rapid organ involvement and small vessel occlusions, confirmed by histopathology and high antiphospholipid antibody levels. The CAPS registry data indicate that commonly affected organs include kidneys, lungs, central nervous system, and the heart, with a high prevalence of lupus anticoagulant and anticardiolipin antibodies (aCL). Current management strategies focus on therapeutic anticoagulation, immunosuppressive therapies like corticosteroids, and adjunct treatments such as plasmapheresis and intravenous immunoglobulin (IVIG). Early use of glucocorticoids and combination therapy has significantly improved outcomes. In life-threatening cases, especially with microangiopathy, experts recommend performing plasma exchange (PE). Patients with associated autoimmune conditions or refractory cases may receive cyclophosphamide (CY) and rituximab while considering PE for treatment. Maintenance of anticoagulation with an appropriate international normalized ratio (INR) is crucial to prevent recurrence. This article reviews the pathogenesis and epidemiology of CAPS. It also examines the current management strategies, and discusses the challenges and controversies associated with these strategies. It hereafter offers recommendations for future management and outlines directions for further research.

## Introduction and background

Antiphospholipid syndrome (APS) is an autoimmune disorder that involves the formation of blood clots in blood vessels and/or complications during pregnancy, occurring in the presence of antiphospholipid antibodies [1,2]. These antibodies include lupus anticoagulant (LA), anticardiolipin antibodies (aCL), and anti-beta-2-glycoprotein I (aβ2GPI) antibodies, which are detected through specific tests such as the enzyme linked immunosorbent assay (ELISA) [2,3,4]. The categorization of APS into two types (primary and secondary) is based on the presence or absence of another autoimmune disease [5]. Primary APS manifests independently without any underlying autoimmune disease. Conversely, secondary APS occurs in conjunction with an autoimmune disorder such as systemic lupus erythematosus (SLE) or occasionally with other autoimmune conditions, infections, drugs, and malignancies [1,2,5,6,7]. The prevalence of APS is estimated at approximately five per 100,000 individuals, with significant differences observed across age, sex, and geographic region [8]. 

The term "catastrophic" was first introduced by Asherson to describe a particularly severe and rapidly progressing complication of APS, thereby distinguishing a distinct subtype that frequently culminates in multiorgan failure and death [1,9,10]. Catastrophic APS (CAPS)_is also known as Asherson's syndrome, honouring Ronald A. Asherson, whose work significantly advanced the understanding of this condition [6,9]. Patients with CAPS typically share several key characteristics: a) clinical signs of involvement in multiple organs (commonly three or more) developing rapidly; b) histopathological evidence of occlusions in multiple small vessels; and c) laboratory confirmation of high titers of antiphospholipid antibodies (aPLA) [11,12]. Although this complication occurs in less than 1% of APS patients, it’s potentially deadly outcomes underscore its critical importance in modern clinical medicine [13,14]. CAPS often presents after precipitating factors such as the cessation or poor compliance of anticoagulation therapy, surgical interventions, minor procedures, or infections [15].

This study offers a comprehensive overview of the pathophysiology and mechanisms of CAPS, analyzes the clinical features and diagnostic criteria associated with CAPS, and evaluates the effectiveness of current treatment approaches. Additionally, the study aims to identify potential gaps in current management practices and propose future directions for improving patient outcomes.

## Review

Pathogenesis

Antiphospholipid antibodies (aPLAs), particularly aβ2GPI antibodies, are central to the pathogenesis of CAPS [1,16]. These antibodies are essential for diagnosing CAPS and are often associated with microthrombosis [6,9,17]. When aPLAs activate endothelial cells (ECs), they increase the production of cell adhesion molecules (e.g., E-selectin, ICAM-1, and VCAM-1, release inflammatory cytokines (e.g., interleukin-1β (IL-1β) and IL-6), and enhance prostacyclin metabolism [18,19]. aβ2GPI antibodies reduce the anticoagulant function of β2GPI on EC surfaces, promoting a prothrombotic state [20,21]. The binding of aβ2GPI to β2GPI activates ECs and induces nuclear factor kappa-light-chain-enhancer of activated B cells (NF-κB) nuclear translocation [21]. This activation increases the production of inflammatory cytokines (e.g., tumour necrosis factor alpha (TNF-α), IL-1β, IL-6, IL-8) and procoagulant factors, including tissue factor and plasminogen activator inhibitor-1 (PAI-1), facilitating leukocyte and platelet adhesion to the endothelium. This process contributes to diffuse microvasculopathy, characterized by microvascular thrombosis and multiorgan failure [16].

aPLAs disrupt natural anticoagulants like protein C, protein S, antithrombin, and annexin A5. They inhibit thrombosis [1,20,22]. aβ2GPI antibodies can recognize peptides on β2GPI that mimic bacterial and viral antigens, suggesting that infections might trigger APS and CAPS. The interaction of β2GPI with Toll-like receptors (TLRs) on ECs, especially TLR-4, activates intracellular signalling pathways involving IL-1 receptor-associated kinases (IRAK) and MyD88, leading to NF-κB activation and a robust inflammatory response [20,23,24]. Various infectious agents, including dengue, typhoid fever, and respiratory pathogens, have been identified as potential triggers for CAPS [25]. These infections stimulate aβ2GPI antibody production through molecular mimicry, where bacterial or viral antigens resemble β2GPI [16]. This immune response can generate cross-reactive aβ2GPI antibodies. Systemic inflammatory response syndrome (SIRS) is a common consequence of infection in CAPS, presenting with acute respiratory distress syndrome (ARDS), encephalopathy, and cardiac dysfunction [26].

Both septic and non-septic triggers can initiate SIRS, highlighting the connection between sepsis and CAPS. Sepsis mechanisms, including excessive cytokine release and microvascular thrombosis, are also evident in CAPS [14, 27]. The recognition of lipopolysaccharides (LPS) by TLRs, particularly TLR-4, in sepsis parallels the inflammatory activation induced by aβ2GPI antibodies in CAPS [6].

In CAPS, widespread micro thromboses resemble the pathology seen in disseminated intravascular coagulation (DIC), characterized by extensive intravascular fibrin formation, vessel occlusion, tissue necrosis, and organ failure [6, 28]. Common triggers for both DIC and CAPS include infections, trauma, surgery, obstetric complications, and malignancy [15]. ECs release proinflammatory cytokines (IL-1, IL-6, TNF-α) in response to aPLAs or infections, activating a cytokine network [19]. This response disrupts protein C an anti-coagulant and other anticoagulant pathways, contributing to a hypofibrinolytic state. Like sepsis, CAPS involves neutrophil sequestration and aggregation in renal blood vessels and extensive infiltration in multiple organs, leading to multiorgan involvement [6,14,27].

**Figure 1:**
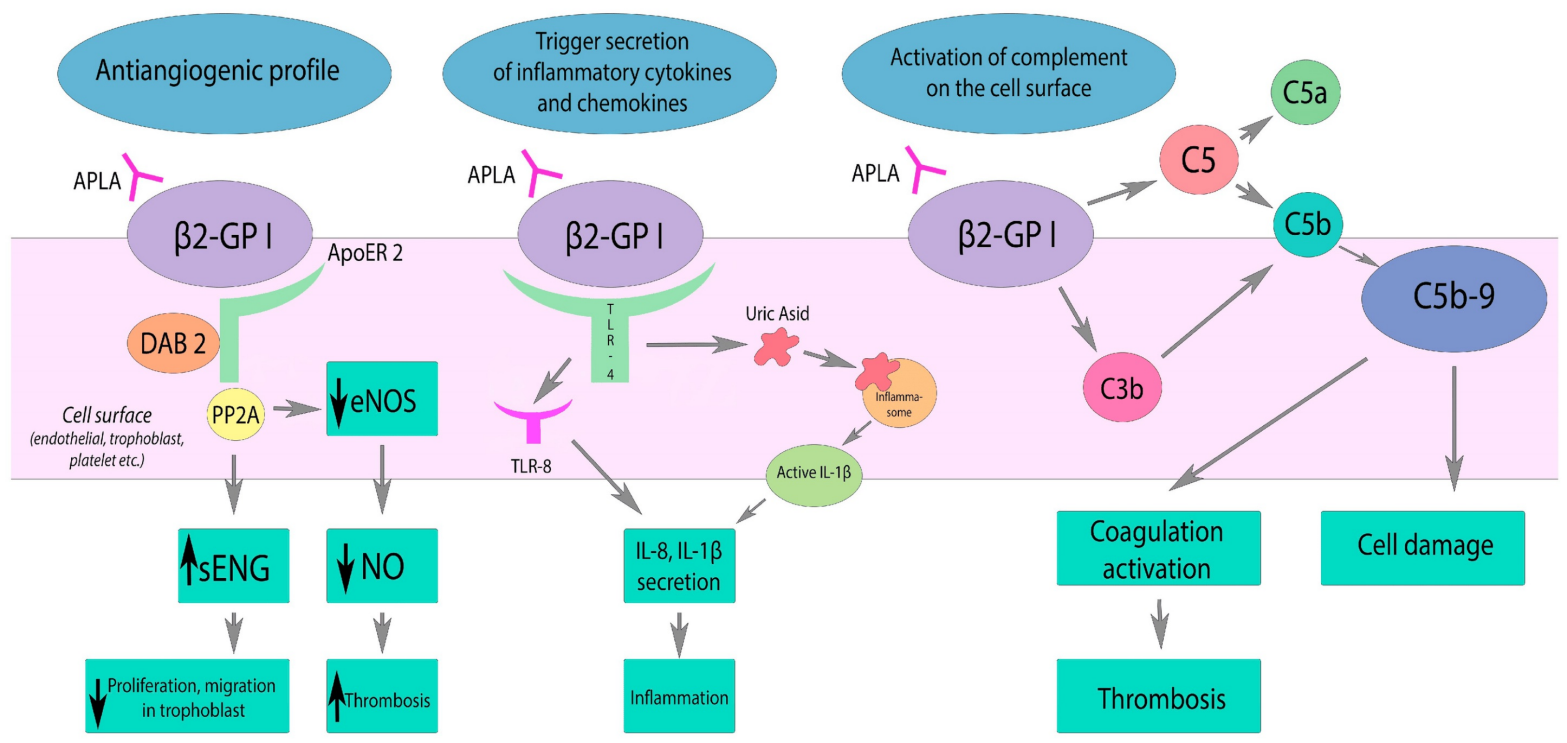
Effects of aPLA on the complement system, inflammation, and vascular tone. Black arrows indicate the direction of change: downward for a decrease and upward for an increase [6]. aPLA diminishes eNOS activity through its interaction with ApoER2, resulting in decreased NO production. This reduction in NO leads to impaired vasodilation and endothelial dysfunction. Additionally, aPLA triggers the activation of TLR and inflammasome pathways, promoting the release of inflammatory cytokines and chemokines. On the cell surface, aPLA also activates the complement system, culminating in coagulation activation and cell damage due to C5b-9 deposition. β2GPI: Beta-2-glycoprotein I, ApoER2: Apolipoprotein E2 receptor; DAB2: Disabled-2; PP2A: Protein phosphatase 2A; eNOS: Nitric oxide synthase; sENG: Soluble endoglin; TLR: Toll-like receptor; TLT-8: Toll-like receptor 8; aPLA: Antiphospholipid antibodies; NO: Nitric oxide

Kitchens postulated the idea of 'thrombotic storm', a term describing the syndrome's rapid and severe clinical manifestations [29]. This condition arises from a genetic predisposition (the first hit) and an environmental trigger (the second hit), such as infection or surgery, which initiates thrombus formation [29,30]. The SIRS in CAPS is driven by excessive cytokine release (IL-1, IL-6, TNF-α), contributing to both local thrombosis and widespread systemic effects [19,26]. In 2003, Raschi et al. further investigated the connection between aPLAs, specifically aβ2GPI, and EC activation [24]. In APS, the binding of aβ2GPI antibodies to ECs, monocytes, and platelets activates NF-κB and initiates an extracellular complement cascade. This activation enhances vascular cell adhesion molecule expression and TNF-α release, promoting thrombosis [24]. Complement activation, particularly through the classical pathway, plays a crucial role in CAPS, leading to platelet activation, adhesion, and aggregation. The mechanism by which this takes place involves the cell surface trophoblasts, ILs, and nitric oxides (NOs) as depicted in Figure 1 [6].

Epidemiology

Unlike classic APS, which typically involves single venous or arterial occlusions, CAPS is characterized by simultaneous small-vessel occlusions affecting multiple organs [13]. The primary source of data on CAPS is the international CAPS registry, established by the European Forum on Antiphospholipid Antibodies. This registry compiles clinical, laboratory, and therapeutic data on reported cases globally, and as of September 2013, it documented 433 patients corresponding to 469 episodes of CAPS , this increased to 500 reported cases by 2016 and 584 by 2022 [3,9,31,32]. However, determining the incidence and prevalence of CAPS is challenging due to its rarity and diagnostic complexity. CAPS affects a subset of APS patients, often following a triggering event such as infection, surgery, or the withdrawal of anticoagulation therapy [13,14]. In 49.1% of patients, the initial clinical manifestation of APS was CAPS [3].

Additionally, mutations in complement regulatory genes have been implicated as potential risk factors [33]. The registry data indicate that CAPS generally presents in younger patients and has a higher incidence among those with SLE [9].

The mortality rate for CAPS has historically been high. Early studies reported mortality rates as high as 50% [9]. However, more recent data suggest a significant improvement in survival rates due to advancements in treatment protocols [13]. Between 2001 and 2005 , the mortality rate decreased to 33.3%, compared to 53% before 2000. A 2016 study of the CAPS registry reported a mortality rate of 37%, encompassing both primary CAPS and SLE-associated CAPS, with as low as 25% reported in recent publications [31,34]. This reduction in mortality is attributed to the earlier recognition of the syndrome and the more frequent use of aggressive combination therapies, including anticoagulation, corticosteroids, PE, and intravenous immunoglobulins (IVIG) [3,13]. An analysis of two distinct periods within the CAPS registry revealed that the mean age at diagnosis decreased from 39.4 years in the earlier period (pre-2000) to 34.4 years in the later period (2001-2005).

In a study conducted by Bucciarelli et al., the primary cause of death was identified in 71.1% of patients [13]. The most frequent cause was cerebral involvement (27.2%), followed by cardiac involvement and infections (19.8% each), multiple organ failure (17.3%), pulmonary involvement (9.9%), and abdominal involvement (4.9%). Autopsies were performed on 50.9% of the patients, revealing micro thrombosis (84.5%), infarcts (53.4%), large vessel thrombosis (19%), pulmonary embolism (12.1%), nonbacterial thrombotic endocarditis (27.6%) [13]. Another study carried out by Gebhart et al. found that the kidneys were most frequently affected (73%), primarily with renal insufficiency (74.5%) [35]. Proteinuria occurred in 25%, haematuria in 12.7%, and hypertension in 22.2%. Pulmonary issues affected 58.9%, including ARDS (37.1%) and emboli (24.9%). CNS involvement was seen in 55.9%, with encephalopathy (40.2%) and stroke (35.2%). Cardiac problems were noted in 49.7%, primarily heart failure (42.1%) and myocardial infarction (27.8%). Skin issues included livedo reticularis (42.3%) and skin necrosis (23.5%). Peripheral vascular involvement was 36.2%, mainly venous (69.2%). Less frequently affected were the intestines (24%), spleen (16.7%), adrenal glands (10.6%), pancreas (7.2%), retina (5.8%), and bone marrow (3.1%) [35].

Clinical Presentation and Diagnosis

CAPS is a severe variant of APS, hence an understanding of the diagnostic criteria for APS which is distinct from that of CAPS is important. The diagnosis of APS has both clinical and laboratory criterions which have been recently updated. The new American College of Rheumatology (ACR) and the European Alliance of Associations for Rheumatology (EULAR) criteria for diagnosing APS include certain clinical and laboratory entities previously considered as “non-criteria manifestations” of APS including skin, kidney, heart, and haematologic complications of the syndrome in the old 2006 Sapporo criteria. The classification criteria as well as the differences between the old Sapporo criteria and new ACR/EULAR criteria are listed in Table 1.

**Table 1:** Main differences between 2006 revised Sapporo and 2023 ACR/EULAR classification criteria for APS [8] * in the setting of high- or low-thrombotic risk profiles (diagnostic weight varying). ** proven histologically or clinically (diagnostic weight varying). aPLA: Antiphospholipid antibodies; aCL: Anticardiolipin antibodies; aβ2GPI: Anti-beta-2-glycoprotein I; GPL: IgG phospholipid unit; MPL: IgM phospholipid unit; LA: Lupus anticoagulant

	2006 Revised Sapporo	2023 ACR/EULAR
Classification	≥1 clinical criteria AND ≥1 laboratory criteria	≥3 points from clinical domains AND ≥3 points from laboratory domains
Clinical criteria	Clinical criteria: 1. Vascular thrombosis: ≥1 clinical episode of arterial, venous, or microvascular thrombosis in any tissue or organ 2. Pregnancy morbidity	Clinical domains: 1. Macrovascular—venous thromboembolism * 2. Macrovascular—arterial thromboembolism * 3. Microvascular ** 4. Obstetric 5. Cardiac valve 6. Haematology
Included in the APS criteria		
Heart valve disease	No	Yes
Livedo racemosa	No	Yes
Thrombocytopenia	No	Yes
Nephropathy	No	Yes
Neurological manifestations	No	No
Pulmonary haemorrhage	No	Yes
Adrenal haemorrhage	No	Yes
Laboratory criteria		
Persistent positivity (at 12 weeks)	Yes	Yes
Timeline of aPLA positivity and clinical criteria	Within 5 years of clinical criterion	Within 3 years of clinical criterion
Threshold of aCL and/or aβ2GPI	aCL > 40 GPL/MPL units, or >99th percentile aβ2GPI > 99th percentile	aCL or aβ2GPI: Moderate: 40–79 units High: ≥80 units
Antibodies for laboratory criteria		
Positive LA	Yes	Yes
IgG aCL or aβ2GPI	Yes	Yes
IgM and/or aβ2GPI	Yes	Yes (not sufficient if isolated)

The clinical manifestations of CAPS can be categorized into those caused by ischemic organ damage and those resulting from systemic inflammatory responses due to cytokine release [14,36]. Patients typically present with multiple thrombotic occlusions, microangiopathic anaemia, and thrombocytopenia, complicating differentiation from other thrombotic microangiopathies. CAPS predominantly affects women, many of whom present in their fourth decade of life, although it can occur across all age groups [3,37]. Identifiable precipitating factors are present in over half of CAPS cases, including infections, surgical procedures, malignancies, withdrawal of anticoagulation, low international normalized ratio (INR) levels, obstetric complications, certain drugs, and SLE flares [31]. Infections, particularly from Gram-negative bacteria, are the most common triggers in children, whereas haematological diseases and malignancies are more frequent in adults [38]. Neoplasms, associated with a thrombophilic state, are the second most common precipitating factor, with increased thrombosis risk in cancer patients attributed to mechanisms such as blood flow stasis, upregulation of thrombophilic substances, and the effects of chemotherapy and central venous devices [31,39].

CAPS manifests through widespread thrombotic microangiopathy, significantly impacting organs such as the kidneys, which were most frequently affected (73%), primarily with renal insufficiency (74.5%). Proteinuria occurred in 25%, haematuria in 12.7%, and hypertension in 22.2%. Pulmonary issues affected 58.9%, including (ARDS) (37.1%) and emboli (24.9%). CNS involvement was seen in 55.9%, with encephalopathy (40.2%) and stroke (35.2%). Cardiac problems were noted in 49.7%, primarily heart failure (42.1%) and myocardial infarction (27.8%). Skin issues included livedo reticularis (42.3%) and skin necrosis (23.5%). Peripheral vascular involvement was 36.2%, mainly venous (69.2%). Less frequently affected were the intestines (24%), spleen (16.7%), adrenal glands (10.6%), pancreas (7.2%), retina (5.8%), and bone marrow (3.1%) [35].

The International Congress on aPLA's classification criteria for CAPS requires the involvement of three organs, systems, or tissues; simultaneous manifestations within a week; small vessel occlusion confirmed histologically in at least one organ; and documented presence of aPLA on two occasions at least 12 weeks apart as succinctly explained in Table 2 [3].

**Table 2:** Preliminary criteria for the classification of CAPS [3] CAPS: Catastrophic antiphospholipid syndrome; aCL: Anticardiolipin antibodies

Criteria	Description
1	Evidence of involvement of three or more organs, systems, and/or tissues
2	Development of manifestations simultaneously or in less than one week
3	Confirmation by histopathology of small vessel occlusion in at least one organ or tissue
4	Laboratory confirmation of the presence of antiphospholipid antibodies (lupus anticoagulant and/or aCL)

A diagnosis of definite CAPS is made when a patient meets all four criteria listed in Table 1.

Potential CAPS is diagnosed when one of the criteria is absent/incomplete such as:

1. Criteria 2, 3, and 4 are present, but only two organs or systems are involved in the pathological process, instead of three and more;

2. Criteria 1, 2, and 3 are observed, but there is no laboratory confirmation of the presence of aPLA;

3. Criteria 1, 2, and 4 are present; or

4. Criteria 1, 3, and 4 are present, but clinical manifestations develop within 1 month, despite anticoagulant therapy [3,9].

In children, CAPS is more commonly linked to peripheral venous thrombosis (37% compared to 23% in elderly adults) and heart failure (37% compared to 19%). Conversely, in elderly adults, CAPS is more frequently associated with arterial thrombosis (33% compared to 16% in children) and renal involvement (87% compared to 71%) [37,40].

CAPS patients frequently display symptoms linked to cytokine storms and microangiopathic phenomena, such as livedo reticularis and Raynaud’s syndrome, with purpura observed less often [41]. Severe HELLP (haemolysis, elevated liver enzymes, and low platelets) syndrome is a significant feature during pregnancy, a period already predisposed to thrombotic events [42]. The clinical presentation and triggers of CAPS vary widely depending on the patient's age and whether the syndrome is primary or secondary [31]. In children, CAPS is more often associated with peripheral venous thrombosis and heart failure, while in older adults, arterial thrombosis and renal issues are more common [37]. Recurrence of CAPS is rare, occurring more than 30 days after apparent remission, documented in less than 5% of cases [13]. Biological data from the CAPS Registry indicates a high prevalence of LA in 83% of confirmed cases, anticardiolipin (aCL) IgG in 81%, aCL IgM in 49%, aβ2GPI IgG in 78%, and antiβ2GPI IgM in 40% [33,37]. Additionally, 58% of patients exhibit low plasma levels of C3 and C4 complement fractions, though their correlation with clinical presentation, thrombotic state, or mortality is inconsistent. Thrombotic microangiopathy is biologically evident in approximately one-third of cases [37, 43].

Current management strategies

Where applicable, initial management should include trigger identification and elimination. This includes pregnancy termination and treating infections, such as a SARS-Cov-2, which has a similar coagulopathy disorder to APS and can lead to catastrophic results [6]. The treatment of CAPS revolves around two primary strategies: therapeutic anticoagulation to manage thrombotic events and additional therapies such as plasmapheresis, IVIG, and immunomodulatory agents to suppress the cytokine cascade [2]. Combination therapy involving anticoagulation, corticosteroids, IVIG, and/or PE is widely accepted. IVIG, in particular, has beneficial effects through its fragment crystallizable (FC) receptor, reducing pathological antibody synthesis and increasing clearance while also suppressing cytokines, modulating T-cell activity, and inhibiting complement activation [23]. IVIG is typically administered at a dose of 0.4 g/day/kg for five days but should be used cautiously due to risks of acute renal failure and thromboembolic events, especially in patients where anticoagulation must be stopped due to bleeding [2,3].

The primary focus of CAPS treatment involves therapeutic anticoagulation to manage thrombotic events. Additional therapies such as plasmapheresis, IVIG, and immunomodulatory agents are also employed to suppress the cytokine cascade [37,44]. The combination therapy of anticoagulation, corticosteroids, IVIG, and/or PE is widely accepted as the standard treatment for CAPS [37,45]. IVIG, in particular, has shown beneficial effects by decreasing the synthesis of pathological antibodies, increasing their clearance, suppressing cytokines, and modulating T-cell activity [34,45]. However, IVIG must be administered with caution due to potential side effects, including acute renal failure and thromboembolic events, especially in patients who cannot continue anticoagulation due to bleeding complications [23,37,45]). For patients diagnosed with thrombotic APS, maintaining anticoagulant therapy with an appropriate INR above 2.0 is essential as a secondary measure to prevent thrombosis [46,47]. This is crucial because recent reviews have identified that discontinuation of anticoagulation or achieving a low INR were triggering factors in 8% of CAPS episodes [47]. Consequently, healthcare providers managing patients with APS should carefully consider the clinical scenarios in which anticoagulant therapy might need to be paused, such as during surgical procedures, biopsies, or dental extractions [47,48]. A study conducted by Espinosa and Cervera revealed that among those treated with anticoagulants, 63.1% recovered, compared to only 22.2% of those not receiving anticoagulant therapy (p<0.0001; OR: 5.98; 95% CI: 2.84-13.80) [49]. The survival rates did not show a statistically significant difference based on the type of anticoagulant used, with unfractionated heparin used in 61% of episodes, oral anticoagulants in 42%, and low-molecular-weight heparin in 13% [49].

Glucocorticoids are used for their anti-inflammatory properties, inhibiting excessive cytokine responses. Early use of glucocorticoids is recommended, with initial doses tailored to clinical manifestations, followed by a gradual tapering [50,51]. PE is indicated in life-threatening CAPS conditions, particularly with microangiopathy features. PE involves removing large quantities of plasma and replacing it with fresh-frozen plasma or albumin [13,45]. The American Society for Apheresis recommends PE with grade 2C evidence for CAPS. Replacement fluid choice remains debated, but a combination of plasma and albumin is suggested to minimize side effects. PE duration is typically three to five days, dictated by clinical response [49].

Cyclophosphamide (CY) is recommended for CAPS patients with SLE features but not for primary APS patients [13]. CAPS registry analysis indicated CY's beneficial effect on SLE-CAPS patients, while it worsened outcomes in primary CAPS patients [3,52]. CY may be used in patients with high aPLA titers to prevent rebound after PE or IVIG treatment, with a recommended dose of 0.5-1 g/m2 [13,16,52].

Rituximab, a chimeric monoclonal antibody against CD20 on B-cells, is approved for several conditions, including B-cell non-Hodgkin lymphoma, rheumatoid arthritis, and autoimmune diseases [53]. In an open-label Phase II trial, rituximab was relatively safe for APS patients and showed benefits in controlling manifestations like thrombocytopenia, skin ulcers, and cognitive dysfunction [53,54]. An analysis of 441 CAPS registry patients found that rituximab was used in 4.6% of cases, mainly as a second-line therapy due to clinical deterioration or poor initial response [54] Among these patients, 75% survived a median follow-up of 9.5 months, suggesting rituximab's potential role in refractory and relapsing CAPS, although the optimal dose remains unknown [54,55].

Eculizumab, a humanized monoclonal antibody against complement C5, prevents the generation and activation of proinflammatory molecules C5a and C5b-9 [56,57]. It is approved for conditions like paroxysmal nocturnal haemoglobinuria and atypical haemolytic uremic syndrome [56,58]. Experimental models indicate that interrupting complement C5a-C5a receptor interaction prevents CAPS complications. Despite limited case reports, eculizumab is considered a last resort medication for relapsing CAPS [56-59].

Other therapeutic options, such as defibrotide, have been clinically used in very few refractory CAPS cases. Defibrotide reduces procoagulant activity and enhances the fibrinolytic properties of vascular ECs [14]. While only two cases of refractory CAPS treated with defibrotide were reported-one resulting in death and the other in complete remission-further studies are needed to establish its efficacy [60]. Additionally, prostacyclin, a potent inhibitor of platelet aggregation and a vasodilator, and fibrinolytics like streptokinase and urokinase theoretically interfere with clotting but lack proven efficacy in CAPS treatment [61].

Challenges and controversies

Recurrent episodes of CAPS are uncommon [59]. Refractory CAPS refers to patients who do not respond to the standard triple therapy of anticoagulants, corticosteroids, and PE or IVIG [8,62,63]. A study conducted by Bucciarelli et al. revealed that relapsing CAPS is characterized by clinical and haematological recurrence after at least 30 days of remission [2]. Factors such as antinuclear antibodies, LA, coexisting lupus, and age over 36 are associated with a higher likelihood of relapse [13]. Data from the CAPS registry indicate that relapsing episodes are rare, occurring in only 3% of patients, with infections and incomplete anticoagulation being common triggers [3,9,64]. Incidence of CAPS is rare and the diagnoses challenging [65]. Relapsing CAP is rarer, hence further study on understanding the cause of the rarity is a systematic study that will need its own dedicated exhaustive research/literature. During relapses, the brain, kidneys, heart, and lungs are frequently affected, and laboratory features of microangiopathic haemolytic anaemia (MHA), such as schistocytes, are prevalent [3]. Treatment often includes anticoagulants and corticosteroids, with additional therapies like PE, IVIG, CY, and rituximab used in some cases. The mortality rate for relapsing CAPS remains high at 33% [3,63].

Diagnosing CAPS is complex due to its rapid onset and non-specific clinical presentation. The 2002 Task Force on CAPS established criteria for definite and probable CAPS with high sensitivity and specificity, further refined with diagnostic algorithms developed at the 13th International Congress on Antiphospholipid Antibodies in 2010 [66]. Despite these efforts, diagnosing CAPS in acute settings remains challenging, with many cases not fitting neatly into the established criteria [63,64]. Retrospective data from the CAPS registry provide the bulk of epidemiological information, but these data are susceptible to biases, including publication bias and heterogeneity in patient cohorts [13,45,64,66].

Interpreting aPLA test results is critical to diagnosing CAPS [67]. False positives can occur due to infections or inflammatory conditions, and anticoagulant treatment can also affect test outcomes [64,67]. Conversely, aPLA levels can be falsely negative during acute thrombotic events. CAPS diagnosis typically relies on double testing of aPLA 12 weeks apart and histological evidence of microvascular thrombosis [64,67]. However, approximately half of CAPS cases occur in patients without a prior history of APS, complicating the diagnostic process [68]. New therapeutic strategies such as rituximab and eculizumab have shown promise for refractory and recurrent CAPS. However, the condition's rarity limits the availability of robust data on their efficacy [69]. The McMaster RARE-Best practices clinical guideline suggests using classification criteria for diagnosis and emphasizes the importance of aPLA positivity [45]. Biopsies, although highly specific, are challenging to perform in acute settings. Consequently, empirical treatment based on clinical suspicion is often necessary, balancing potential benefits and risks until a definitive diagnosis can be confirmed histologically [45,64].

Case report analysis 

In a case report by Strakhan et al., a 36-year-old female presented with decreased responsiveness, profound weakness, and visual disturbances [58]. She also presented with severe hypertension, confusion, and dehydration, alongside lab findings, including elevated white blood cell count, reduced platelet count, and metabolic derangements. Initial investigations revealed multiple organ involvement, with the patient experiencing non-ST segment myocardial infarction (NSTEMI), intraretinal haemorrhage, and acute brain changes. Elevated lactate dehydrogenase levels and the presence of schistocytes on a peripheral blood smear indicated MHA, a feature of CAPS [9,29]. Despite initial plasmapheresis, sustained improvement was not achieved, prompting further investigation into her coagulation profile and immune status. A renal biopsy confirmed thrombotic microangiopathy, consistent with CAPS pathology [32]. The fluctuating nature of LA antibodies observed in this patient post-plasmapheresis reinforces the importance of repeated antibody testing to confirm the diagnosis [34]. The patient's lack of response to standard therapies such as plasmapheresis and corticosteroids, along with challenges in maintaining therapeutic anticoagulation, led to considering eculizumab. This decision was informed by case reports highlighting eculizumab's efficacy in refractory CAPS [70]. The patient showed significant clinical improvement with eculizumab, including stabilized renal function, normalized lactate dehydrogenase levels, and resolution of brain haemorrhagic foci [58].

Another case was reported by del Carpio-Orantes et al., a 52-year-old male with peripheral vascular ulcers and chronic postphlebitic syndrome [71]. The patient presented with acute abdominal pain, ileus, pneumatosis intestinalis, and free fluid, leading to the diagnosis of intestinal and omental necrosis due to segmental mesenteric thrombosis. Initial and subsequent surgeries were required, along with intensive care support due to multiorgan failure. A thrombophilia workup revealed weakly positive aCL and histopathological findings suggested a thrombotic process. Consultation with Dr Ricard Cervera suggested CAPS, leading to treatment with enoxaparin, methylprednisolone, and intravenous IgG. The patient showed significant improvement, emphasizing the potential for favourable outcomes with appropriate treatment. This case supports that peripheral vascular ulcers and postphlebitic syndrome can be manifestations of undiagnosed primary APS [3,13,16]. It also highlights the importance of recognizing and aggressively managing CAPS to reduce morbidity and mortality [3,16].

Recommendations for future management practice

It is essential to emphasize early diagnosis and intervention to improve care for patients with CAPS. Enhanced recognition of CAPS symptoms among healthcare providers is crucial, given the rapid progression and severe outcomes associated with the syndrome. Education and training programmes should be implemented to ensure clinicians can identify CAPS early and differentiate it from other thrombotic microangiopathies. Timely diagnosis is pivotal for initiating appropriate treatment strategies and improving patient outcomes [3]. Optimizing anticoagulation therapy is a crucial recommendation for managing CAPS. Patients diagnosed with thrombotic APS should maintain therapeutic anticoagulation with an INR above 2.0 to prevent thrombosis. Healthcare providers must carefully consider the clinical scenarios that may require the temporary discontinuation of anticoagulation, such as surgical procedures, biopsies, or dental extractions [46,47]. Ensuring continuity of anticoagulation therapy can significantly reduce the risk of CAPS episodes triggered by low INR levels or cessation of anticoagulation.

The use of combination therapy, including corticosteroids, IVIG, and PE, should be standardized as part of CAPS treatment protocols. IVIG has shown beneficial effects by reducing pathological antibody synthesis, increasing antibody clearance, and modulating immune responses. However, it must be used cautiously due to potential side effects, including acute renal failure and thromboembolic events [23,45]. PE, particularly in life-threatening cases of CAPS with microangiopathy features, involves removing large quantities of plasma and replacing it with fresh-frozen plasma or albumin and is recommended with a grade 2C evidence by the American Society for Apheresis [49]. Tailoring immunosuppressive therapy based on individual patient characteristics, especially in those with SLE, is essential. CY has demonstrated beneficial effects in SLE-CAPS patients but may worsen outcomes in primary CAPS patients [16,52]. Therefore, CY should be reserved for patients with high antiphospholipid antibody titers and those with SLE features to prevent rebound after PE or IVIG treatment. Additionally, the use of rituximab, a monoclonal antibody targeting CD20 on B-cells, has shown promise in controlling CAPS manifestations and may be considered as a second-line therapy in patients who do not respond to initial treatments [53,54].

Establishing a comprehensive care networks and more specialized centers for CAPS management similar to the ones found in major hospitals like john Hopkins in the US and Kings college Hospital in the UK can significantly improve patient outcomes. These centers should offer multidisciplinary care, including rheumatologists, haematologists, nephrologists, and other specialists, to address the multiorgan involvement characteristic of CAPS. Collaborative research and clinical practice efforts can facilitate the development of standardized treatment protocols and enhance the understanding of CAPS pathophysiology, ultimately leading to improved management and survival rates for patients with this rare and severe condition [3].

Strengths of the review

This review offers a comprehensive and detailed examination of CAPS, effectively synthesizing information from multiple case reports and authoritative sources. One of its primary strengths lies in the meticulous analysis of individual case studies, such as those presented by Strakhan et al. and del Carpio-Orantes et al., which provide real-world insights into the clinical presentation, diagnostic challenges, and therapeutic responses of CAPS patients [58,71]. By highlighting specific patient outcomes and the efficacy of various treatment modalities, the review underscores the importance of personalized and evidence-based approaches in managing this complex syndrome. The review emphasizes the necessity of a multidisciplinary approach, involving rheumatologists, haematologists, and critical care specialists, which is crucial for the effective management of CAPS due to its multiorgan involvement. Also, the review’s focus on the evolving understanding of CAPS pathophysiology and the subsequent advancements in treatment protocols. It draws attention to the importance of maintaining therapeutic anticoagulation and the role of combination therapy, including corticosteroids, IVIG, and PE. The inclusion of newer therapeutic options like eculizumab and rituximab for refractory cases illustrates a forward-looking perspective, aligning with the latest research and clinical practices. This thorough approach provides a valuable resource for healthcare professionals seeking to update their knowledge and improve patient outcomes through evidence-based interventions.

Limitations of the review

Despite its comprehensive nature, the review does have several limitations that warrant consideration. One notable limitation is the reliance on case reports and retrospective analyses, which, while informative, may not provide the generalizability and statistical robustness of larger, controlled studies. The specificity of individual patient responses highlighted in case reports, such as those by Strakhan et al. and del Carpio-Orantes et al., may not be universally applicable, limiting the broader applicability of the findings [58,71]. Furthermore, the review does not adequately address the potential biases inherent in case report literature, such as publication bias, where only positive outcomes tend to be reported, potentially skewing the perception of treatment efficacy. Another limitation is the relatively scant discussion on the long-term outcomes and follow-up of patients with CAPS. While the review provides detailed accounts of acute management and short-term responses, it lacks depth in exploring the chronic management strategies and long-term prognoses of CAPS patients. Additionally, the review could benefit from a more critical appraisal of the existing diagnostic criteria and the challenges associated with early and accurate diagnosis of CAPS. The complexity of differentiating CAPS from other thrombotic microangiopathies is acknowledged, but further elaboration on diagnostic advancements and the role of emerging biomarkers would enhance the review’s utility for clinical practitioners. 

## Conclusions

CAPS is a rare but severe variant of APS characterized by rapid onset and widespread small-vessel thrombosis leading to multiorgan failure. Despite its rarity, CAPS presents significant clinical challenges due to its high mortality rate and complex pathophysiology involving immune, thrombotic, and inflammatory processes. Advances in understanding the mechanisms of CAPS have improved diagnostic criteria and treatment protocols, contributing to better patient outcomes. However, despite these advancements, CAPS remains a critical condition requiring immediate and aggressive treatment. Early recognition, prompt and aggressive intervention, and ongoing research into novel therapeutic targets are essential to improving the prognosis for patients with this devastating syndrome. The multidisciplinary approach, integrating rheumatology, haematology, and critical care expertise, will continue to be pivotal in the management of CAPS, ensuring that advances in research translate into meaningful clinical outcomes for affected patients.
